# Healthcare quality measures in implementation research: advantages, risks and lessons learned

**DOI:** 10.1186/s12961-022-00934-y

**Published:** 2022-12-07

**Authors:** Allison M. Gustavson, Hildi J. Hagedorn, Leah E. Jesser, Marie E. Kenny, Barbara A. Clothier, Mark Bounthavong, Princess E. Ackland, Adam J. Gordon, Alex H. S. Harris

**Affiliations:** 1 Veterans Affairs Health Services Research and Development Center for Care Delivery and Outcomes Research, Minneapolis Veterans Affairs Healthcare System, 1 Veterans Drive, Mail Code#152, Minneapolis, MN 55417 USA; 2 Department of Psychiatry, University of Minnesota School of Medicine, Minneapolis, MN 55455 USA; 3 Health Economics Resource Center, VA Palo Alto Healthcare System, Palo Alto, CA 94025 USA; 4 Department of Medicine, University of Minnesota, Minneapolis, USA; 5 Vulnerable Veteran Innovative PACT (VIP) Initiative; Informatics, Decision-Enhancement, and Analytic Sciences Center (IDEAS), Salt Lake City Veterans Healthcare System, 500 Foothill Drive, Salt Lake City, UT 84148 USA; 6 Program for Addiction Research, Clinical Care, Knowledge and Advocacy (PARCKA), University of Utah School of Medicine, Department of Internal Medicine, University of Utah School of Medicine, Salt Lake City, UT 84148 USA; 7 Center for Innovation to Implementation, Palo Alto Veteran Affairs Healthcare System, Palo Alto, CA 94025 USA; 8 Department of Surgery, Stanford University School of Medicine, Stanford, CA 94305 USA

**Keywords:** Implementation science, Healthcare quality measures, Stakeholders, Implementation research

## Abstract

Implementation studies evaluate strategies to move evidence-based practices into routine clinical practice. Often, implementation scientists use healthcare quality measures to evaluate the integration of an evidence-based clinical practice into real-world healthcare settings. Healthcare quality measures have standardized definitions and are a method to operationalize and monitor guideline-congruent care. Implementation scientists can access existing data on healthcare quality measures through various sources (e.g. operations-calculated), or they can calculate the measures directly from healthcare claims and administrative data (i.e. researcher-calculated). Implementation scientists need a better understanding of the advantages and disadvantages of these methods of obtaining healthcare quality data for designing, planning and executing an implementation study. The purpose of this paper is to describe the advantages, risks and lessons learned when using operations- versus researcher-calculated healthcare quality measures in site selection, implementation monitoring and implementation outcome evaluation. A key lesson learned was that relying solely on operations-calculated healthcare quality measures during an implementation study poses risks to site selection, accurate feedback on implementation progress to stakeholders, and the integrity of study results. A possible solution is using operations-calculated quality measures for monitoring of evidence-based practice uptake and researcher-calculated measures for site section and outcomes evaluation. This approach provides researchers greater control over the data and consistency of the measurement from site selection to outcomes evaluation while still retaining measures that are familiar and understood by key stakeholders whom implementation scientists need to engage in practice change efforts.

## Background

The call for rapid translation of evidence-based interventions into clinical practice has increased demand for implementation research across a multitude of disciplines, clinical settings and healthcare systems [1]. Implementation science seeks to understand and address the challenges of applying evidence-based practices in real-world settings to optimize the quality and delivery of healthcare [1]. Implementation scientists focus on understanding the current use of evidence-based practices and evaluating approaches to promote adoption of evidence-based practices into routine healthcare.

One way to evaluate *how much* and *how well* an evidence-based clinical practice is implemented in a real-world healthcare setting or system is to use existing healthcare quality measures [2]. Health quality measures are calculated using standardized definitions and methods to operationalize guideline-congruent care [3, 4]. Healthcare quality measures are often used in implementation research as they capture information on provider(s) adherence to clinical practice guidelines and the impact of provider services on patient health outcomes [5]. Importantly, these measures are familiar to and viewed as relevant and accurate by key stakeholders (e.g. clinicians, leadership) whom implementation scientists need to engage in practice change efforts [6].

Most healthcare quality measures are calculated using existing administrative data (e.g. claims) and/or clinical data (e.g. information in the electronic medical records [EMR]). To calculate healthcare quality measures, many organizations employ teams of skilled data scientists to convert existing administrative and clinical data into quality measure data. Often, healthcare quality measures are made publicly available by or for other organizations such as health departments, hospital associations or the Centers for Medicare and Medicaid Services (CMS). Healthcare quality measures used by many healthcare systems and payors include 30-day unplanned readmissions to the hospital, discharge rates from the hospital to the community, infection rates, median time to treatment, healthcare experiences and satisfaction with care, and access to care [3]. Other healthcare quality measures capture the number of patients who receive evidence-based care (numerator) over the number of patients likely to benefit (denominator). For example, the proportion of patients with an opioid use disorder who receive evidence-based medication treatment is a quality concept operationalized into a quality measure by the Veterans Health Administration (VHA) [7], CMS and other professional organizations [8].

As such, implementation scientists may access and use healthcare quality measures by obtaining the data extracted and calculated by others (e.g. healthcare system operations, CMS or health departments) through spreadsheets, dashboards or publicly available data. Alternatively, implementation scientists may directly calculate healthcare quality measures themselves based on standard definitions (i.e. researcher-calculated). While many advantages exist to using healthcare quality measures in implementation research, a better understanding of the risks and benefits regarding the different methods to extract the data is essential to designing implementation studies that inform clinical care and contribute to generalizable knowledge. Failure to account for, and acknowledge, the risk–benefit trade-offs of using operationally calculated vs researcher-calculated quality measures data during any phase of an implementation study can considerably, and negatively, impact the ability to successfully interpret results. The purpose of our perspective article is to describe the advantages, risks and lessons learned when using different sources of healthcare quality measure data in site selection, implementation monitoring and outcome evaluation. We will use an illustrative example of one implementation study to guide this perspective.

## Illustrative example: implementation study overview

The protocol and results of the Advancing Pharmacological Treatments for Opioid Use Disorder (ADaPT-OUD) implementation study are described elsewhere [7, 9]. As a brief overview, medication for OUD (MOUD; including buprenorphine, methadone and naltrexone) is an evidence-based treatment for persons with OUD [10]. However, substantial variability in adoption of MOUD exists across the VHA [9, 11]. The purpose of the ADaPT-OUD study was to increase utilization of MOUD in low-adopting VHA facilities through external facilitation (e.g. coaching and mentoring by the research team) [7, 9]. VHA operations calculates and tracks a MOUD quality measure defined as the proportion of patients with an OUD diagnosis within each VHA facility who receive MOUD (MOUD/OUD ratio). The MOUD/OUD ratio is made available quarterly on a dashboard for use by clinicians and operational leaders to view progress in MOUD adoption [12]. For the ADaPT-OUD study, we chose and received approval to use the operations-calculated MOUD/OUD ratio quality measure for site selection, implementation monitoring and outcome evaluation. We will refer to the MOUD/OUD ratio as our operations-calculated healthcare quality measure when illustrating how the choice not to calculate the measure ourselves impacted of the ADaPT-OUD implementation study.

## Impact of healthcare quality measure use on site selection

Often, implementation scientists use healthcare quality measures to identify and target low adopters of a clinical intervention [13–15] and then evaluate the effectiveness of specific strategies in supporting the increased adoption of that clinical intervention. In the ADaPT-OUD study, the primary advantage of using an operation-calculated healthcare quality measure (MOUD/OUD ratio) was that it facilitated communication with stakeholders, particularly facility leaders and clinicians, who were already familiar with the metric and their facility’s standing on that metric. We used this operations metric for initial site selection of low-adopting VHA facilities, which we operationalized as facilities in the bottom quartile based on the facilities MOUD/OUD ratio. The risk in using operations-calculated quality measures versus researcher-calculated metrics to determine site eligibility is that operational leadership may institute changes to the definition of the measure or discontinue calculating the measure mid-project [16]. This can lead to inaccurate conclusions about the level of change achieved by facilities during the study or difficulties in continued tracking of performance over time.

**Fig. 1:**
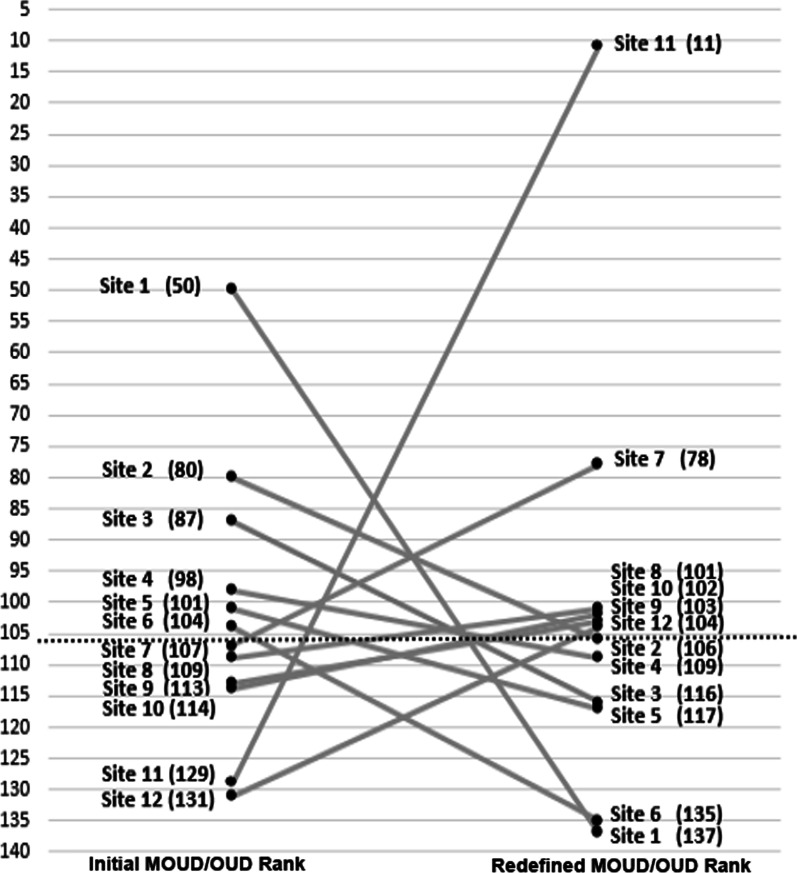
Change in rank and performance on the MOUD/OUD healthcare quality measure before and after redefinition. Change in rank of the MOUD/OUD measure for the 12 VHA facilities (i.e., sites) that moved from or to the bottom quartile (out of a total of 140 facilities). The parentheses indicate the site rank out of 140 facilities, with lower ranks indicating better MOUD/OUD ratios. The area below the dashed line denotes the lowest-performing quartile. Facilities in the lowest quartile, defined by the lowest MOUD/OUD ratios, were classified as low-adopting. None of the facilities depicted were in our intervention arm

To illustrate this example, approximately 9 months after ADaPT-OUD site selection, we found that the baseline MOUD/OUD ratio we had extracted from operations data at initial site selection no longer matched the historical data available in operations dashboards. Upon further investigation, we found that the definition of the numerator (number of patients prescribed MOUD) and denominator (number of patients with OUD diagnosis) constituting the MOUD/OUD ratio were changed by operations. Subsequently, the redefinition of the MOUD/OUD ratio was applied retroactively to the previous four quarters of available operations dashboard data such that the original MOUD/OUD ratio for our selected sites was no longer available. This means that VHA operations applied a new definition for the MOUD/OUD ratio to the prior year (i.e., during our site selection) and overwrote the administrative data we had previously captured and documented for site selection. From an operations perspective, the change in MOUD/OUD ratio calculations was prompted by a need to improve the accuracy in identifying patients with OUD by excluding patients with the “opioid use, unspecified” diagnosis code (affecting the MOUD/OUD denominator) and correctly capture the receipt of MOUD by including MOUD prescribed by providers outside of the VHA healthcare system (affecting the MOUD/OUD numerator). As a result of the MOUD/OUD redefinition, six sites initially operationalized as low adopters at the time of site selection were in a higher MOUD/OUD quartile than previously recorded. An additional six sites originally in the better-performing three quarters were reassigned to the lowest quartile. Figure 1 shows the 12 sites that moved in or out of the lowest quartile (defined by the MOUD/OUD ratio) when comparing the historical MOUD/OUD ratio to the newly defined MOUD/OUD ratio. Fortunately, all eight of the intervention sites originally selected for ADaPT-OUD remained in the lowest quartile of MOUD/OUD ratio.

## Impact of healthcare quality measure use on monitoring progress and evaluating outcomes

Healthcare quality measures are also used to monitor implementation progress and evaluate outcomes across multiple sites. In the ADaPT-OUD study, the use of operations-calculated healthcare quality measures in monitoring ADaPT-OUD progress and evaluating outcomes helped create a common language between researchers and stakeholders (e.g., clinicians, leadership). This connection and understanding of the MOUD/OUD healthcare quality measure allowed researchers to provide stakeholders relevant feedback on their progress and formulate individualized plans to achieve adoption of evidence-based practice. However, as with site selection discussed above, the continued risk in using operations-calculated healthcare quality measures for implementation monitoring and evaluation of outcomes is that operations may change the healthcare quality measure definition over time.

To illustrate with the ADaPT-OUD study, our primary method of monitoring progress in uptake of MOUD and measure of implementation success (i.e. primary study outcome) was the change in MOUD/OUD ratio from baseline to 12 months between control and intervention sites. If we had not become aware of the redefinition of the MOUD/OUD ratio, then we may have erroneously calculated healthcare quality change values that included two different measures. The use of incorrect data in interpretations of study results has implications for future research and organizational policies regarding the use of resource-intensive implementation strategies to promote clinical adoption of evidence-based practice. Our study outcomes show that our implementation strategy was effective in increasing MOUD in these low-adopting sites [7]. However, if we had used two different measures to calculate outcomes, then we may have erroneously deemed our implementation strategy ineffective, thereby missing an opportunity to identify and spread an effective implementation strategy.

## Summary of lessons learned

A key lesson learned was that relying solely on operations-calculated data for healthcare quality measures during an implementation study poses risks to the identification of low-adopting sites, accurate feedback on progress to stakeholders and the integrity of study results (Table 1). Thus, using a combination of operations-calculated quality measures for implementation monitoring and a researcher-calculated measure for site section and outcome evaluation may afford researchers more control over the data and consistency in the measurement from site selection to outcomes evaluation and, importantly, still allow for critical stakeholder engagement in adopting evidence-based practices. To achieve the benefits of both operations-calculated quality measures (easier communication with facility stakeholders) and research-calculated metrics (enhanced control over data integrity and consistency), more intensive data calculation and monitoring processes would be required, which has implications for study budgets and resource allocation of personnel. This increased data management, monitoring and calculation by researchers requires careful consideration and understanding of the specifications of a healthcare quality measure (e.g. variable definition, time frame of data collection). Moreover, this process requires additional validation steps to ensure that the measures accurately represent the construct and are reliable throughout the study period [17]. Additionally, a scenario analysis can be performed to test the robustness of the study results across a range of potential values such as operations- and researcher-calculated measures [18]. If use of operations-calculated healthcare quality measures is the only option, then another key consideration is planning regular contact with the stewards of the measure to ensure full and timely awareness of any anticipated changes and allow time to plan responses to changes.

**Table 1:** An overview of the advantages, risks and lessons learned regarding different methods of healthcare quality measure data collection for use in implementation research

Healthcare quality measure	Advantages	Disadvantages/risks	Lessons learned
Pre-calculated (by operations, health departments, federal agencies)	• Less intensive for researchers to extract • Creates a common language for discussion with and feedback to stakeholders	• May be redefined at any time based on operational priorities	• Consider operations-calculated for monitoring implementation progress with stakeholders • Communicate regularly with operations partners to confirm definitions • Modify study budgets and consider research-calculated for site selection and evaluation of outcomes • Perform a scenario analysis using both operations- and researcher-calculated measures
Researcher-calculated	• Increased stability of the measure’s definition	• Higher personnel costs for data extraction and management	

## Conclusions

Implementation studies conducted using healthcare quality measures hold tremendous potential to rapidly impact the delivery of high-value healthcare across complex settings. Yet, research teams must carefully consider and create a plan for monitoring methods of data extraction and use through all phases of the study to ensure the reliability and validity of study results. In the future, this may require operations’ investment or engagement in the implementation study to foster transparency in any changes to definitions of healthcare quality measures. Implementation scientists must also carefully evaluate study budgets to ensure adequate funding is allotted towards multimodal data collection and extraction of healthcare quality measures. Increased awareness and attention to the appropriate use of healthcare quality measures in implementation science is needed to make advancements in the field and improve healthcare.

## Data Availability

Deidentified data can be obtained by contacting the corresponding author.

## Abbreviations

EMR: Electronic medical record
VHA: Veterans Health Administration
ADaPT-OUD: Advancing Pharmacological Treatments for Opioid Use Disorder
OUD: Opioid use disorder
MOUD: Medication treatment for OUD
